# Study on Blood Biomarkers of Subjective Cognitive Decline

**DOI:** 10.1002/alz70856_104231

**Published:** 2025-12-24

**Authors:** Shuhua Ren, Xiaoxie Mao, Qi Huang, Fang Xie, Yihui Guan

**Affiliations:** ^1^ Huashan Hospital, Shanghai, China; ^2^ School of Medicine, Xiamen University, Xiamen, Fujian, China; ^3^ Department of Nuclear Medicine & PET Center, Huashan Hospital, Fudan University, Shanghai, Shanghai, China; ^4^ Huashan Hospital, Fudan University, Shanghai, Shanghai, China; ^5^ Department of Nuclear Medicine and PET Center, Huashan Hospital, Fudan University, Shanghai, China

## Abstract

**Background:**

This study aimed to investigate the characteristics of “ATN” blood biomarkers in SCD and their relationship with “A” obtained from imaging and cognitive function, and assess whether blood “ATN” can predict longitudinal cognitive changes in SCD patients, offering new insights into early diagnosis biomarkers.

**Method:**

A total of 321 subjects were included, consisting of 246 SCD and 75 normal controls (NC). Neuropsychological testing, 18F‐Florbetapir PET/CT imaging for Aβ deposition, and blood samples for plasma Aβ42, Aβ40, *p*‐Tau181, t‐Tau, and NfL levels were collected. After 2 years, 95 subjects underwent follow‐up cognitive assessments. Independent sample t‐tests analyzed intergroup differences, ROC curves assessed diagnostic performance, and partial correlations explored the relationship between blood biomarkers, Aβ deposition, and cognitive function. The change rate (Δchange rate = (follow‐up ‐ baseline) / baseline) was used to analyze cognitive function changes, and a general linear model examined the correlation between baseline biomarkers and cognitive changes.

**Result:**

Aβ‐positive SCD showed a trend of lower blood Aβ42/Aβ40 ratio compared to Aβ‐negative SCD. Blood *p*‐Tau181 and *p*‐Tau181/Aβ42 were positively correlated with whole‐brain Aβ SUVr (*r* = 0.12, *p* =  0.028; r = 0.15, *p* =  0.005). In SCD, blood *p*‐Tau181 correlated negatively with memory (AVLT‐N5) and positively with executive function (STT‐A). Blood NfL negatively correlated with memory and language functions, but positively with executive functions (STT‐A and STT‐B). Baseline blood NfL predicted cognitive decline in SCD (β = ‐0.37, *p* < 0.05).

**Conclusion:**

Blood *p*‐Tau181/Aβ42 is correlated with Aβ deposition in SCD, while NfL predicts cognitive decline in SCD, highlighting its potential for early diagnosis.

